# The Mitigating Effect of Combined Glucocorticoids with Immune Checkpoint Inhibitors on Lymphocyte Activation Gene‐3 and Programmed Death‐1 Expression

**DOI:** 10.1002/eji.70033

**Published:** 2025-08-11

**Authors:** Smadar Gertel, Ari Polachek, Victoria Furer, Tali Ofir Dovrat, Chen Avaky, Adi Broyde, Hila Nochimovitz, Ori Elkayam

**Affiliations:** ^1^ Department of Rheumatology Tel‐Aviv Sourasky Medical Center Tel‐Aviv Israel; ^2^ The Gray Faculty of Medical and Health Sciences Tel‐Aviv University Tel‐Aviv Israel; ^3^ Department of Rheumatology Rabin Medical Center Petach Tikva Israel

**Keywords:** glucocorticoids, Immune checkpoint inhibitors, LAG‐3, synovial monocytes, T cells

## Abstract

Cancer immunotherapy with immune checkpoint inhibitors (ICI) shows promising therapeutic efficacy but can cause immune‐related adverse events (irAEs). Glucocorticoids (GCs) are commonly employed with ICI to mitigate irAEs. We had found previously that GCs upregulate significantly the inhibitory molecule, lymphocyte activation gene‐3 (LAG‐3) in peripheral blood and synovial fluid mononuclear cells (PBMCs and SFMCs, respectively). Here, we investigated the effect of GCs combined with ICI on LAG‐3 and programmed death‐1 (PD‐1) expression in SFMCs of 32 inflammatory arthritis patients and PBMCs of 15 healthy controls. GC+Pembrolizumab (PEM, anti‐PD‐1) induced IL‐10 and suppressed IFN‐γ, TNF‐α, and IL‐17A mRNA expressions compared with PEM alone in PBMCs and SFMCs. PBMC proliferation was markedly inhibited by GC+PEM (3.5 ± 0.7%, *p *< 0.0006) compared with PEM alone (26.2 ± 6.5%). GC+PEM increased the CD4^+^LAG‐3^+^ T cells (4.9±1.2%, *p *< 0.03) compared with PEM alone (0.9 ± 0.3%), but did not affect CD4^+^PD‐1^+^ T cells. The effect of the drugs on synovial cells revealed that GC+PEM remarkably increased the CD14^+^LAG‐3^+^ cells in SFMCs (10.4 ± 2.0%, *p *< 0.0001) compared with PEM alone (0.6 ± 0.2%), but not the CD14^+^PD‐1^+^ cells. Thus, GC combined with ICI might exhibit contrasting activity via upregulation of CD4^+^LAG‐3^+^ T and CD14^+^LAG‐3^+^ cells in circulation and synovial milieu, respectively, possibly interfering with the ICI activity.

## Graphical abstract

Mechanism of GCs combined with ICI may induce LAG‐3, an inhibitory molecule expression (A) Immune checkpoint inhibitors (ICIs) are a class of drugs that work by blocking inhibitory molecules on immune cells (B) Glucocorticoids (GCs) are used to manage the immune‐related adverse events following ICI therapies (C) Concomitant GCs with ICI therapies can induce peripheral CD4^+^LAG‐3^+^ T cells and synovial monocytes CD14^+^LAG‐3^+^ cells.

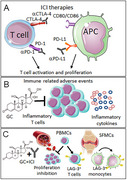

## 1 Introduction

Immune checkpoint inhibitors (ICIs) are immunomodulatory antibodies that enhance T‐cell function by blocking negative regulatory receptors on T cells. The primary targets for immune checkpoint inhibition have included cytotoxic T‐lymphocyte‐associated antigen 4 (CTLA‐4), programmed death‐1 (PD‐1), and programmed death ligand‐1 (PD‐L1). ICIs have led to remarkable success in patients with various malignancies [1, 2, 3]. While ICIs hold great promise, their nonspecific immune activation can give rise to autoimmune‐like or inflammatory symptoms known as immune‐related adverse events (irAEs) [4, 5]. The proposed mechanisms of action include increased T cell activity against antigens in tumors and in healthy tissues, inflammatory cytokine production, elevated levels of pre‐existing autoantibodies, and enhanced complement‐mediated inflammation. By means of these processes, ICI increases immune activity and may induce irAEs, leading to a wide range of clinical manifestations ranging from mild to fatal forms [6]. irAEs are diverse in nature and can affect any organ systems, resulting in rheumatic, dermatological, pulmonary, gastrointestinal, cardiovascular, renal, endocrine, and neurological toxicities [7, 8]. The reported prevalence of ICI‐induced irAEs ranges from 15% to 90% [9, 10] and may cause severe morbidity, treatment discontinuation, and even death [11]. Immune suppression with systemic corticosteroids (glucocorticoids [GCs]) has become the first‐line treatment to mitigate irAEs [12]. As such, GCs represent the mainstay of treatment for moderate‐to‐severe irAEs, with other conventional synthetic disease‐modifying anti‐rheumatic drugs (DMARDs), like methotrexate [MTX], and biologic DMARDs (e.g., anti‐TNF or anti‐IL‐6 receptor and others) recommended for second‐line therapy [13, 14, 15].

GCs act at multiple levels to suppress T‐cell effector responses, specifically, inhibition of dendritic cell antigen presentation, inhibition of cytokine production, and modulation of various T‐cell populations [16]. Although GCs can successfully mitigate irAEs, their immunosuppressive activity on treatment outcomes must be taken into consideration. While GCs inhibit inflammation through the regulation of anti‐inflammatory mediators, several studies showed that combining ICIs with GCs to mitigate irAEs can negatively impact the clinical outcomes of cancer patients [17, 18, 19]. Contrarily, others did not find any negative impact on treatment outcomes related to the effectiveness of combined GCs and ICI therapy [20, 21]. GCs were shown to upregulate PD‐1 and CTLA‐4 with concomitant downregulation of T cell activation in vitro [22, 23]. In addition to PD‐1 and CTLA‐4, other inhibitory molecules were shown to be capable of inhibiting the immune response. Among such inhibitory molecules, lymphocyte activation gene‐3 (LAG‐3) reportedly plays a pivotal role in autoimmunity, tumor immunity, and chronic infections [24, 25, 26].

LAG‐3 is a type I transmembrane protein with structural similarities to CD4 that binds to MHC class II molecules and negatively regulates T cell function, thereby providing immune suppression [27]. We have demonstrated before that the anti‐inflammatory and immunosuppressive effects of GCs are mediated, at least in part, through significant upregulation of the inhibitory molecule LAG‐3 on peripheral blood and synovial fluid mononuclear cells (PBMCs and SFMCs, respectively) [28]. Here, we investigated the in vitro effects of GCs and other DMARDs combined with ICI on peripheral and synovial immune cells. We specifically hypothesized that GCs might induce LAG‐3 up‐regulation following co‐culture with GC and ICI.

## 2 Results

### 2.1 GC Combined with PD‐1 Inhibitor (PEM) Induces Inflammatory Gene Expression in Healthy Controls PBMCs

**FIGURE 1:**
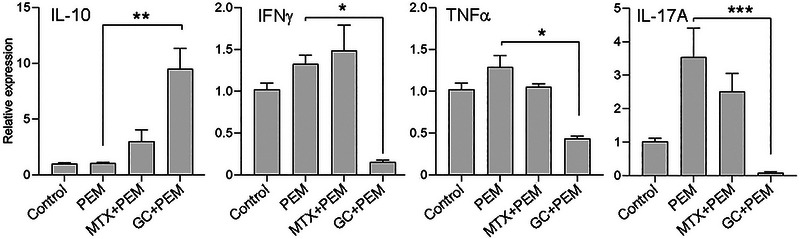
GC with PEM modulates pro‐ and anti‐inflammatory gene expression in PBMCs. Healthy donors’ PBMCs (*n* = 3) were cultured for 72 h in the presence of PEM, MTX+PEM, GC+PEM, or no drug (control). IL‐10, IFN‐γ, TNF‐α, and IL‐17A mRNA expression was determined by real‐time PCR. Data are shown as relative expression, normalized to GAPDH, mean ± SEM, analyzed with the Kruskal–Wallis test followed by Dunn's post hoc comparisons, *p < 0.01, **p < 0.002, ***p < 0.0001.

To analyze the in vitro activity of GC with anti‐PD‐1, pembrolizumab (PEM) on the modulation of gene expression, we examined the potency of PEM alone, GC+PEM, MTX+PEM, and no drug control in healthy donor PBMCs following 72 h of incubation. As shown in Figure 1, the relative expression of the anti‐inflammatory IL‐10 was not modulated by PEM alone compared with no drug control. IL‐10 was significantly upregulated by GC+PEM (9.5 ± 1.8, *p *< 0.002) but not by MTX+PEM (3.0 ± 1.8) compared with PEM alone (1.0 ± 0.07). Neither IFN‐γ nor TNF‐α was significantly modulated by PEM alone compared with no drug control. GC+PEM significantly downmodulated IFN‐γ and TNF‐α expression (0.16 ± 0.02, *p *< 0.01 and 0.43 ± 0.03, *p *< 0.01, respectively) compared with PEM alone (1.3 ± 0.1 and 1.3 ± 0.1, respectively). MTX+PEM did not exhibit similar activity. The inflammatory IL‐17A was upregulated by PEM alone (3.5 ± 0.8) compared with no drug control (1.0 ± 0.1), but it did not reach a level of significance. Consistent with the results of IFN‐γ and TNF‐α, IL‐17A was significantly down‐modulated by GC+PEM (0.08 ± 0.03, *p *< 0.0001) but not by MTX+PEM compared with PEM alone.

### 2.2 GC Alone or Combined with PEM Inhibits PBMCs’ Proliferation with Concomitant Upregulation of CD4^+^LAG‐3^+^ T Cells

**FIGURE 2:**
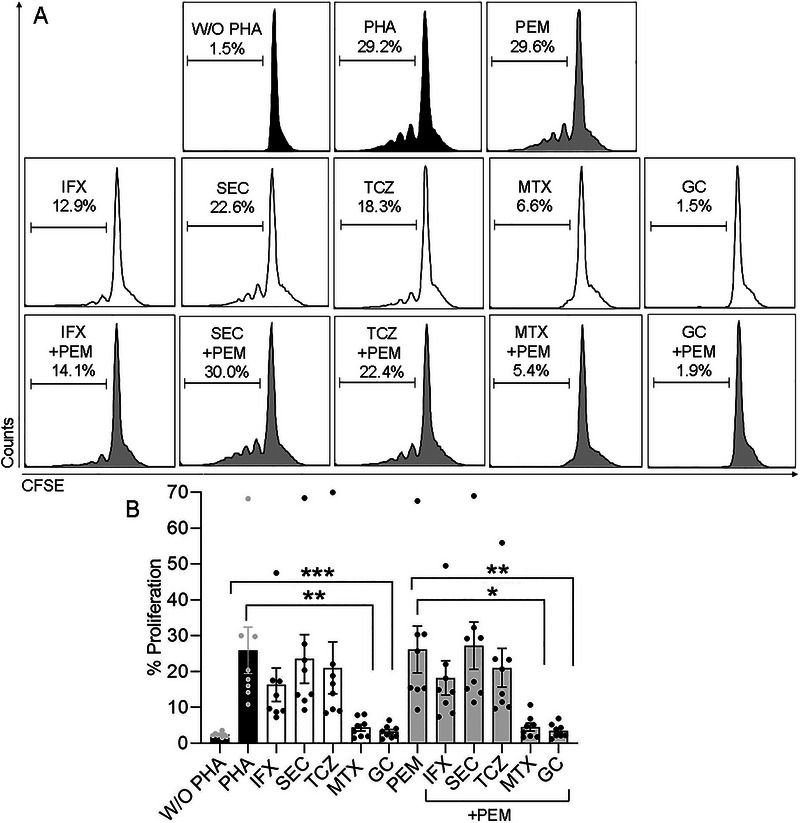
Both GC and MTX alone or combined with PEM inhibit PHA‐induced proliferation in healthy donors’ peripheral blood mononuclear cells (PBMCs). Carboxyfluorescein succinimidyl ester (CFSE)‐labelled PBMCs from healthy donors (*n* = 8) were incubated with or without PHA 5 µg/ml, or PHA with the indicated drugs. The cells were harvested and analyzed after 5 days. (A) Representative plots showing examples of the CFSE dilution assay with PBMCs derived from a healthy subject. The extent of proliferation is shown on the left side of each plot. Upper panel: cells without PHA (no proliferation), with PHA alone (maximal proliferation), or with PEM alone. Middle panel: IFX 10 µg/mL, SEC 10 µg/mL, TCZ 10 µg/mL, MTX 50 ng/mL, and GC 5 µg/mL. Lower panel: PHA with the tested drugs combined with PEM. Percentages indicate the proliferation rate exerted by each drug. (B) Summary of data showing the percentage of proliferation. Comparisons were determined by the Kruskal–Wallis test followed by Dunn's post hoc test. Data are shown as mean ± SEM. *p < 0.002, **p < 0.0006 and ***p < 0.0001. IFX, infliximab; GC, glucocorticoid; MTX, methotrexate; PEM, pembrolizumab; PHA, phytohemagglutinin; SEC, secukinumab; TCZ, tocilizumab; W/O, without.

It is well established that GC inhibits inflammatory reactions by suppression of pro‐inflammatory cytokines and inhibits lymphocyte proliferation. Here, we investigated the effect of the GC and DMARDs alone and combined with PEM on cell proliferation in healthy control PBMCs. As shown in Figure 2, the average extent of the unstimulated PBMCs (no PHA) proliferation rate was 2.3 ± 0.2% (representing the control). The proliferation rate of PHA‐stimulated PBMCs increased to 25.9 ± 6.5%, reflecting maximal proliferation. The PHA‐stimulated PBMCs’ proliferative response in the presence of the tested drugs was then assessed. The drugs' ability to reduce lymphocyte proliferation (when cultured with PHA) was indicated by the reduced percentage of cells showing carboxyfluorescein succinimidyl ester (CFSE) dilutions (Figure 2A,B). PEM alone (26.2 ± 6.5%) had no effect on the proliferation rate compared with PHA alone (Figure 2A, upper panel). As shown in Figure 2A, middle panel, the biologic DMARDs tested as single agent namely: anti‐TNF, infliximab (IFX), anti‐IL‐17A, secukinumab (SEC), and anti‐IL‐6 receptor, tocilizumab (TCZ) did not inhibited the proliferation rate significantly as compared with PHA alone (16.3 ± 4.7%, 23.4 ± 6.8% and 21.0 ± 7.2%, respectively) although IFX inhibited to a greater extent the proliferation rate as compared with SEC and TCZ. In the presence of MTX and GC, a significant inhibition in the proliferation rate (4.4 ± 0.9%, *p *< 0.0006 and 3.2 ± 0.6%, *p *< 0.0001, respectively) as compared with PHA alone was observed (Figure 2A, middle panel + B). As for drug combinations, the biologic DMARDs combined with PEM (IFX+PEM, SEC+PEM, or TCZ+PEM: 18.2 ± 4.8%, 27.1 ± 6.6%, and 21.1 ± 5.4%, respectively) did not significantly affect the proliferation rate compared with PEM alone. In contrast, MTX+PEM and GC+PEM (4.5 ± 1.1%, *p *< 0.002 and 3.5 ± 0.7%, *p *< 0.0006, respectively) significantly reduced the proliferation rate compared with PEM alone (Figure 2A, lower panel + B).

**FIGURE 3:**
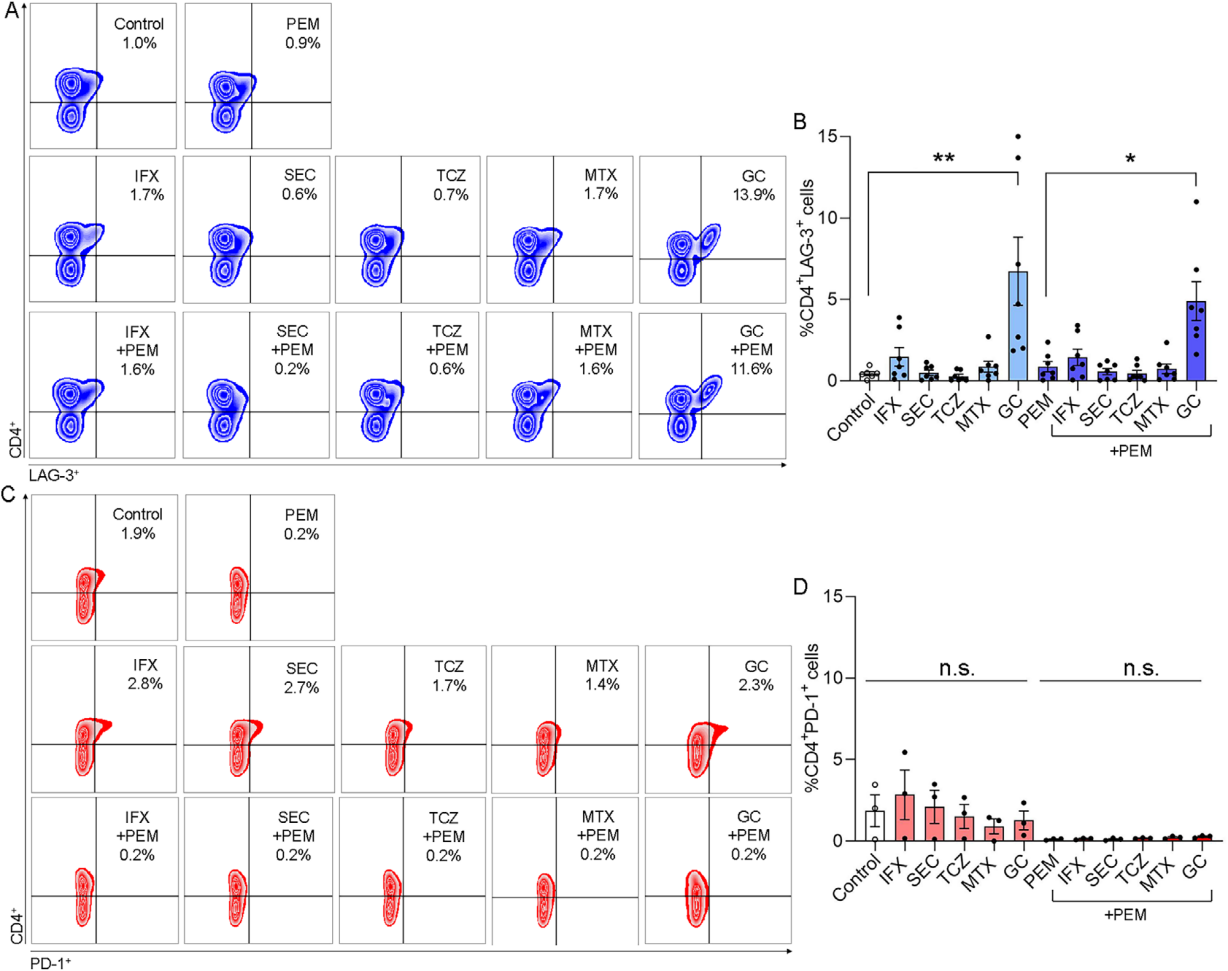
GC alone or combined with PEM promotes LAG‐3 but not PD‐1 on peripheral CD4^+^ T cells. Healthy donor PBMCs were cultured in the presence of PHA 5 µg/mL without any drug (control) or with PEM 10 µg/mL (upper panel). Comparison of the frequencies of CD4^+^LAG‐3^+^ T cells after co‐culture with the following drugs as a single agent: IFX 10 µg/mL, SEC 10 µg/mL, TCZ 10 µg/mL, MTX 50 ng/mL, and GC 5 µg/mL (middle panel) or the tested drugs combined with PEM (lower panel). Cells were cultured for 5 days, and at the end of the culture, they were harvested and analyzed by flow cytometry. (A) Representative frequencies dot plots of CD4^+^LAG‐3^+^ T cells. The upper right quadrant in each plot shows the percentage of CD4^+^LAG‐3^+^ T cells. (B) %CD4^+^LAG‐3^+^ T cells in healthy donor PBMCs (*n* = 7). (C) Representative dot plots of CD4^+^PD‐1^+^ T cells. (D) %CD4^+^PD‐1^+^ T cells in healthy donor PBMCs (*n* = 3). Statistical significance was calculated using the Kruskal–Wallis test with Dunn's multiple comparison test, *p < 0.03, **p < 0.02, and n.s: nonsignificant.

In the same experiments, the effect of the tested drugs on CD4^+^LAG‐3^+^ T cells was analyzed (Figure 3). PEM alone (0.9 ± 0.3%) did not modulate CD4^+^LAG‐3^+^ T cells compared with on drug control (0.4 ± 0.1%). The effect of the drugs as single agents: IFX, SEC, TCZ, MTX, and GC on the CD4^+^LAG‐3^+^ T cells was determined (Figure 3A,B). Nonsignificant changes in the level of CD4^+^LAG‐3^+^ T cells were detected following incubation with IFX, SEC, TCZ, and MTX (1.5 ± 0.6, 0.5 ± 0.2, 0.3 ± 0.2, and 0.9 ± 0.3, respectively), although IFX increased the CD4^+^LAG‐3^+^ T cells as compared with the other biologic DMARDs. GC significantly increased the CD4^+^LAG‐3^+^ T cells compared with the control (6.7 ± 2.1, *p *< 0.02). The results indicate that in the presence of GC, there is an approximately 6 × increase in CD4^+^LAG‐3^+^ T cells than in the control.

Similarly, among the drug combinations, GC+PEM induced significant upregulation of CD4^+^LAG‐3^+^ T cells (4.9 ± 1.2%, *p *< 0.03), whereas MTX+PEM did not modulate them (0.7 ± 0.3%) compared with PEM alone. None of the combinations of biologics DMARDs+PEM could significantly modulate the CD4^+^LAG‐3^+^ T cells (IFX: 1.5 ± 0.6%, SEC: 0.6 ± 0.2%, and TCZ: 0.5 ± 0.2%) compared with PEM alone (Figure 3). Of note, among the combinations of biologic DMARDs, anti‐TNF (IFX)+PEM led to a slight increase in CD4^+^LAG‐3^+^ T cells, although it was not significant compared with PEM alone.

For the analysis of CD4^+^PD‐1^+^ T cells, we observed unremarkable changes in the presence of IFX, SEC, TCZ, MTX, and GC as single agents (Figure 3C) compared with the control (ranging between 0.9% and 2.8%). In the presence of PEM, a PD‐1 inhibitor, as a single agent or combined with DMARDs or GC, the CD4^+^PD‐1^+^ T cells were even at a lower level compared with no‐drug control (ranging between 0.1% and 0.3%). Thus, it seems that upon supplementation of PEM, there is almost no expression of PD‐1 on CD4^+^ T cells. Interestingly, the drug combinations GC+PEM could not impact the relatively low expression of CD4^+^PD‐1^+^ T cells (Figure 3C,D).

To assess the effect of the drugs on CD8 cytotoxic T cells that participate in antitumor response, in a similar set of experiments, we tested the effects of the drugs on proliferation and CD8^+^LAG‐3^+^ T cells (Figure S1). As shown earlier (Figure 2), a similar trend in proliferation inhibition by the drugs can be seen in Figure S1 A+B. PEM alone (43.6 ± 7.2%) did not inhibit the proliferation compared with PHA alone (40.1 ± 7.7%). IFX alone (34.8 ± 2.9%) and IFX+PEM (34.8 ± 2.9%) inhibited the proliferation compared with PHA alone or PEM alone, respectively, but without reaching statistical significance. Both MTX (9.7 ± 2.3%, *p *< 0.03) and GC (9.7 ± 2.1%, *p *< 0.03) alone significantly inhibited the proliferation as compared with PHA alone (Figure S1A,B). The inhibitory effect of MTX+PEM (9.7 ± 2.0%, *p *< 0.03) and GC+PEM (10.0 ± 2.0%, *p *< 0.05) was significantly higher as compared with PEM alone. As for the effect of the drugs on CD8^+^LAG‐3^+^ T cells (Figure S1C,D), PEM alone (0.2 ± 0.1%) did not modulate CD8^+^LAG‐3^+^ T cells compared with control (0.2 ± 0.1%). The effect of the single agents, IFX, increased CD8^+^LAG‐3^+^ T cells (1.9 ± 0.8%) as compared with control, but the effect was nonsignificant, while with MTX (0.6 ± 0.3%), the CD8^+^LAG‐3^+^ T cells remained at a low level. In contrast, GC significantly increased the CD8^+^LAG‐3^+^ T cells as compared with the control (4.3 ± 1.5, *p *< 0.05). Similarly, among the drug combinations, GC+PEM induced significant upregulation of CD8^+^LAG‐3^+^ T cells (6.7 ± 1.3%, *p *< 0.002), whereas IFX+PEM (2.2 ± 0.7%) or MTX+PEM (1.9 ± 0.9%) could not modulate the CD8^+^LAG‐3^+^ T cells compared with PEM alone.

### 2.3 GCs Combined with PEM Induce Anti‐Inflammatory Gene Expression in Synovial Cells

**FIGURE 4:**
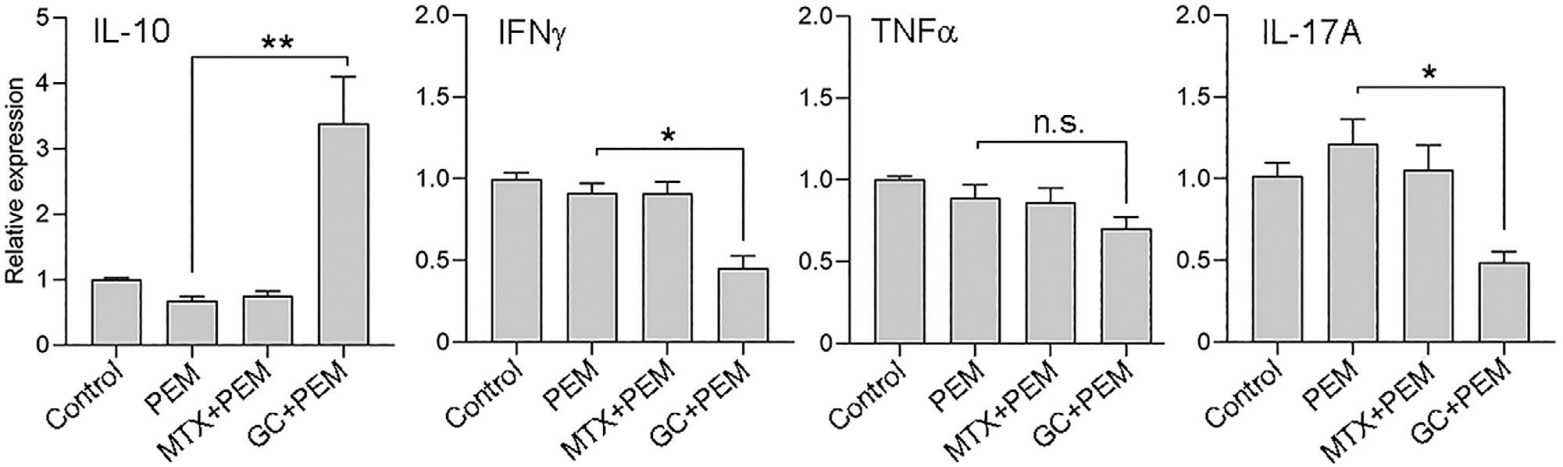
GC with PEM modulates pro‐ and anti‐inflammatory gene expression in SFMCs. SFMCs (*n* = 4) derived from 2 PsA, 1 RA, and 1 SpA patients (except for IL‐17A analysis was performed on (*n* = 3)) were cultured for 72 h in the presence of PEM, MTX+PEM, GC+PEM, or no drug control. IL‐10, IFN‐γ, TNF‐α, and IL‐17A mRNA expression was determined by real‐time PCR. Data are shown as relative expression normalized to GAPDH, mean ± SEM, analyzed with the Kruskal–Wallis test followed by Dunn's post hoc comparisons, *p < 0.005, **p < 0.0001, n.s.: nonsignificant.

Inflammatory synovitis is frequently reported in ICI‐induced rheumatic irAEs. Therefore, we aimed to analyze the effect of ICI combined with GC and other DMARDs used in arthritis management in co‐culture with SFMCs derived from arthritis patients and test their effect on the inflammatory response. The effect of the various drugs on the same genes at the same incubation timeframe that was analyzed in PBMCs was now determined in SFMCs (Figure 4). IL‐10 was not modulated by PEM alone compared with no drug control. IL‐10 was significantly upregulated by GC+PEM (3.4 ± 0.7, *p *< 0.0001) but not by MTX+PEM compared with PEM alone (1.0 ± 0.02). Neither IFN‐γ nor TNF‐α was significantly modulated by PEM alone compared with no drug control. GC+PEM downmodulated IFN‐γ and TNF‐α expression (0.45 ± 0.8, *p* < 0.005 and 0.7 ± 0.1, n.s., respectively) compared with PEM alone (1.0 ± 0.4 and 1.0 ± 0.2, respectively), whereas MTX+PEM did not exhibit similar activity. IL‐17A was significantly downmodulated by GC+PEM (0.49 ± 0.1, *p *< 0.005) but not by MTX+PEM compared with PEM alone (1.2 ± 0.1).

### 2.4 Effect of GC and Various DMARDs Combined with ICI on the Inhibitory Molecules LAG‐3 and PD‐1 Expression in SFMCs

**FIGURE 5:**
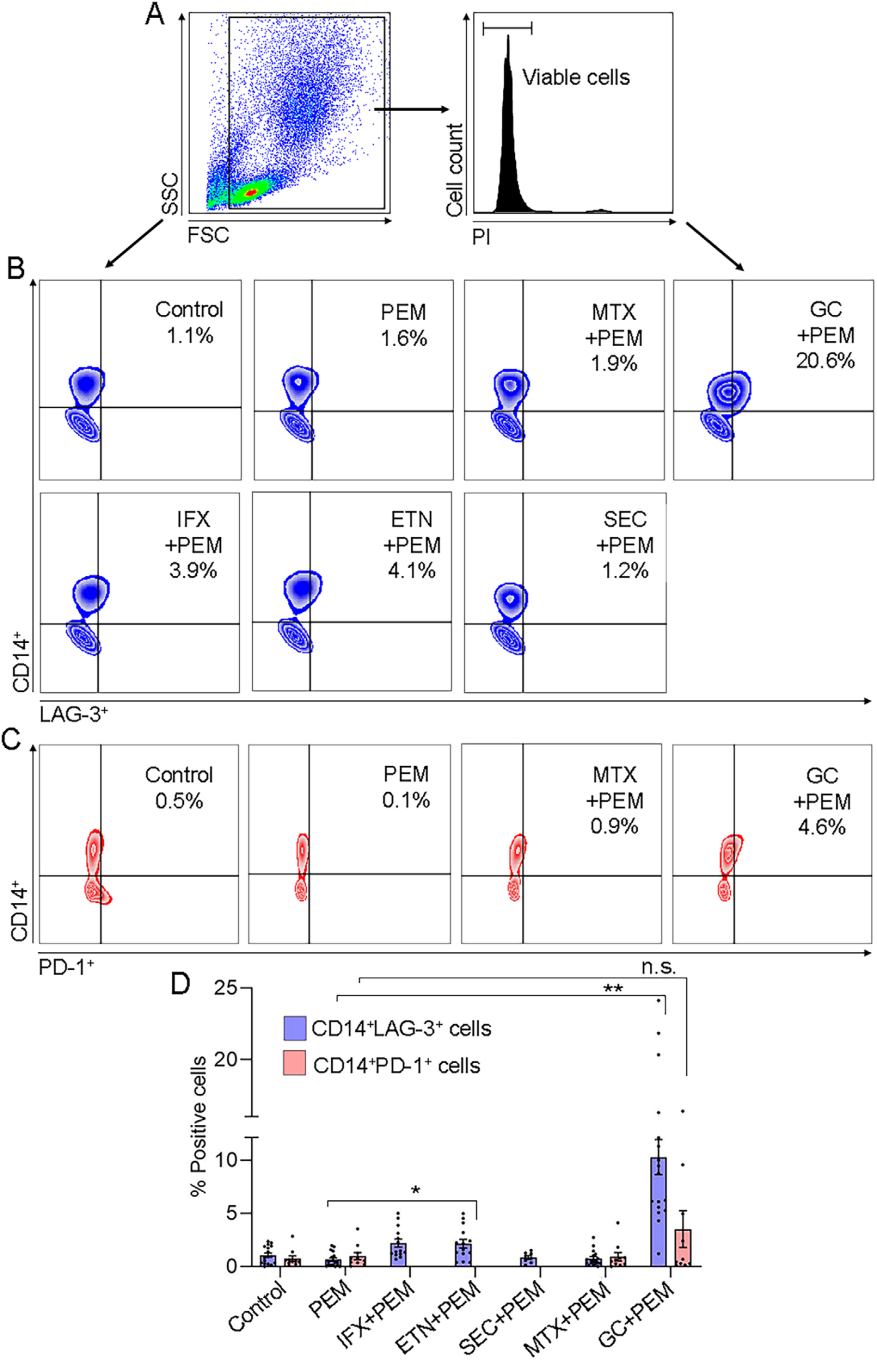
Effect of the GC or DMARDs combined with PEM on LAG‐3 and PD‐1 expression in monocytes derived SFMCs. (A) Dot plots show the gating strategy. Dead cells were excluded, and PI‐negative (viable cells) were analyzed. (B) Representative dot plots of CD14^+^LAG‐3^+^ cells in SFMCs (*n* = 17, 9 PsA, 5 RA, and 3 SpA patients). The upper right quadrant shows the percentage of CD14^+^LAG‐3^+^ cells. (C) Representative dot plots of CD14^+^PD‐1^+^ cells in SFMCs (*n* = 14, 9 PsA and 5 RA patients). The upper right quadrant shows the percentage of CD14^+^PD‐1^+^ cells. (D) The graph shows the frequency of CD14^+^LAG‐3^+^ cells and CD14^+^PD‐1^+^ cells. Each symbol in the graphs represents an individual patient. Data are shown as mean ± SEM. Statistical significance was calculated using the Kruskal–Wallis test with Dunn's multiple comparison test, *p < 0.05, **p < 0.0001, n.s.: nonsignificant.

ICI therapy can affect immunological reactions that lead to synovial inflammation in which activated synovial monocytes participate. Therefore, the effect of combined GC and various DMARDs with ICI on synovial monocytes expressing LAG‐3 and PD‐1 was assessed. As shown in Figure 5A,B, in the control, only a low percentage of CD14^+^LAG‐3^+^ cells (1.1 ± 0.2%) were detected. PEM alone (0.7 ± 0.2%) did not change the CD14^+^LAG‐3^+^ cells compared with the control. The CD14^+^LAG‐3^+^ cells remain low (0.7 ± 0.2%) at the combination of MTX+PEM. There was a significant increase (up to 10‐fold) of CD14^+^LAG‐3^+^ cells with GC+PEM (10.4 ± 1.7%, *p* < 0.0001) compared with PEM alone. As for the combinations of biologic DMARDs with PEM, both anti‐TNFs (IFX and etanercept (ETN))+PEM significantly increased the CD14^+^LAG‐3^+^ cells: 2.2 ± 0.4%, *p* < 0.02, and 2.2 ± 0.4%, *p* < 0.02, respectively, compared with PEM alone, although the increase was less than that following GC+PEM. The combination, SEC+PEM, did not change the CD14^+^LAG‐3^+^ cells compared with PEM alone (Figure 5B–D). The effect of the drugs on LAG‐3 seems to be specific since the proportion of CD14^+^PD‐1^+^ cells was not affected by any of the drugs. The combinations MTX+PEM (1.0 ± 0.4%) or GC+PEM (3.5 ± 1.7%) did not change significantly the percentages of CD14^+^PD‐1^+^ cells compared with PEM alone (1.0 ± 0.3%) (Figure 5C,D).

**FIGURE 6:**
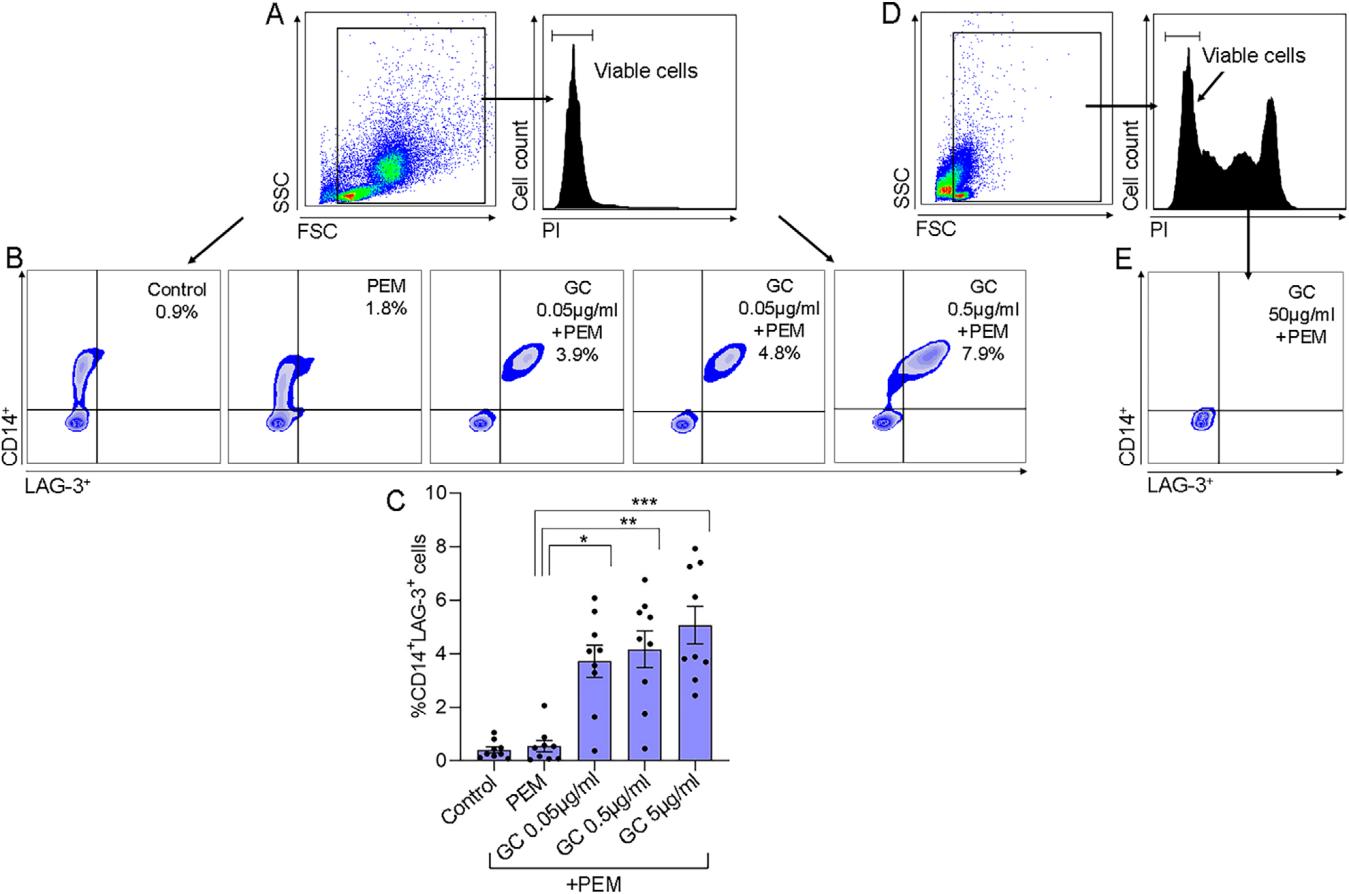
Effect of GC in different doses combined with PEM on CD14^+^LAG‐3^+^ cells derived SFMCs. SFMCs (*n* = 9, 6 PsA, 2 RA, and 1 SpA patients) were co‐cultured with the designated drugs for 4 days and analyzed by flow cytometry. (A) Cells were stained with PI to analyze viable cells (PI negative). (B) SFMCs were co‐cultured in the presence of PEM alone, different GC doses (0.05, 0.5, and 5 µg/mL) with PEM, or no drug (control). Representative dot plots of CD14^+^LAG‐3^+^ cells. (C) The graph shows percentage CD14^+^LAG‐3^+^ cells ± SE. (D) At a concentration of GC 50 µg/mL+PEM, the PI staining shows that a substantial part of the cells are nonviable. (E) A representative dot plot shows  cells that are negative for CD14 and LAG‐3. Statistical significance was calculated using the Kruskal–Wallis test with Dunn's multiple comparison test, *p < 0.02, **p < 0.005, ***p < 0.0006, and n.s.: nonsignificant.

The effect of GC+PEM was evaluated in SFMCs using GC in different doses (Figure 6). The tested concentrations of GC were: 0.05, 0.5, 5, and 50 µg/mL. The impact on CD14^+^LAG‐3^+^ cells was evaluated after 4 days. The combination of GC at the concentrations of (0.05 and 0.5 µg/mL)+PEM significantly increased the %CD14^+^LAG‐3^+^ cells (3.7 ± 0.6%, *p* < 0.02 and 4.2 ± 0.7%, *p *< 0.005, respectively) as compared with PEM alone (0.5 ± 0.2%) (Figure 6B,C). At the concentration of GC 5 µg/mL+PEM, %CD14^+^LAG‐3^+^ cells reached the highest level as compared with PEM alone (5.1 ± 0.7%, *p *< 0.0005). At the highest GC concentration (GC 50 µg/mL+PEM), a sharp decrease in the viable cells was detected, as shown by the PI staining in Figure 6D, and the remaining viable cells were negative for CD14 or LAG‐3, Figure 6E.

**FIGURE 7:**
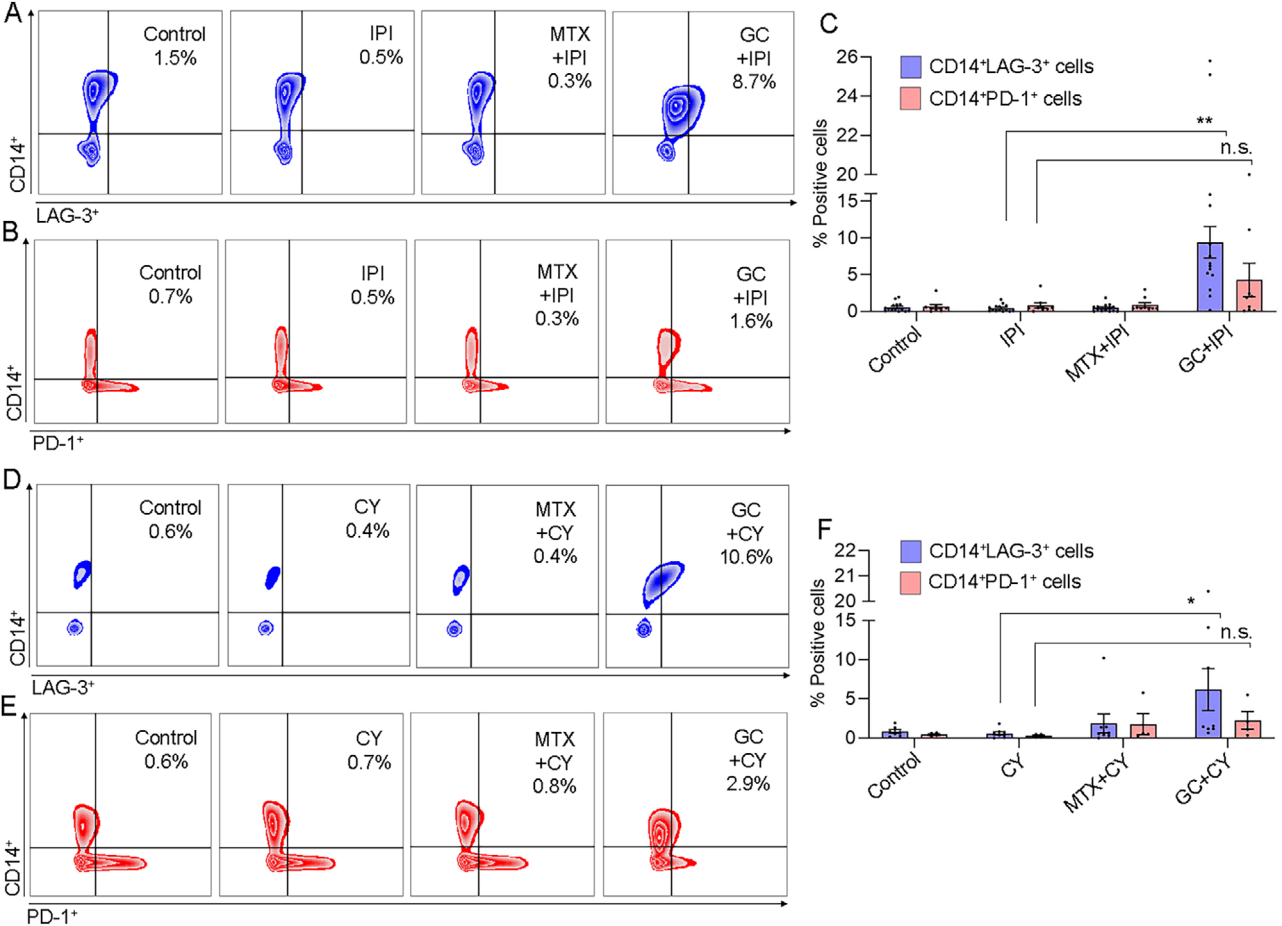
Effect of ipilizumab (IPI) or cyclophosphamide (CY) alone or with MTX or GC on LAG‐3 and PD‐1 expression on CD14^+^ derived SFMCs. The effect of IPI alone or combined with GC or MTX was analyzed on SFMCs (*n* = 14, 8 PsA, 4 RA, and 2 SpA patients) (A–C). The effect of CY alone or combined with GC or MTX was analyzed on SFMCs (*n* = 9, 5 PsA, 2 RA, and 2 SpA patients) (D–F). (C, F) Graphs show frequencies of CD14^+^LAG‐3^+^ cells and CD14^+^PD‐1^+^ cells. Statistical significance was calculated using the Kruskal–Wallis test with Dunn's multiple comparison test. *p < 0.05, **p < 0.0001, n.s.: nonsignificant.

Verification of GC activity combined with other ICI drugs, anti‐CTLA‐4, ipilizumab (IPI), was assessed on LAG‐3 and PD‐1 expressing CD14^+^ synovial cells (Figure 7). For LAG‐3 expression, IPI alone did not change the proportion of CD14^+^LAG‐3^+^ cells compared with control (0.4 ± 0.1% vs. 0.6 ± 0.2%, respectively). In contrast, GC+IPI led to a significant upregulation of 10‐fold in CD14^+^LAG‐3^+^ cells compared with IPI alone (9.4 ± 2.1%, *p *< 0.0001) (Figure 7A‐C), whereas MTX+IPI (0.5 ± 0.1%) did not change the latter population compared with IPI alone. Consistent with the previously observed results of GC combined with PEM on CD14^+^PD‐1^+^ cells, GC+IPI did not significantly change the CD14^+^PD‐1^+^ cells compared with IPI alone (0.8 ± 0.3% vs. 0.7 ± 0.3%, respectively), and also in combination with MTX+IPI (0.9 ± 0.3%), this population remains low (Figure 7B,C).

Cyclophosphamide (CY) is a chemotherapeutic agent used to inhibit the progression of certain tumors and lymphomas [29]. Lastly, we examined the activity of GC combined with CY, an anticancer drug with a different mechanism, on the selected immune populations. As shown in Figure 7D–F, CY alone did not have any effect on CD14^+^LAG‐3^+^ and CD14^+^PD‐1^+^ cells compared with no drug control (for CD14^+^LAG‐3^+^ cells: 0.7 ± 0.3% vs. 0.9 ± 0.3%, respectively, and for CD14^+^PD‐1^+^ cells: 1.4 ± 0.6% vs. 0.8 ± 0.4%, respectively). The activity of GC+CY, however, resulted in a significant increase in CD14^+^LAG‐3^+^ cells (7.9 ± 3.3%, *p *< 0.05) but not in CD14^+^PD‐1^+^ cells (4.4 ± 1.7%) compared with CY alone. The combination MTX+CY had no significant effect on both populations (CD14^+^LAG‐3^+^ cells, 2.4 ± 1.6%, and CD14^+^PD‐1^+^ cells, 1.9 ± 0.8%).

## 3 Discussion

Immune‐related AEs (irAEs) frequently occur during treatment with ICIs. Administration of immunosuppressive drugs, such as GCs, is widely used for managing ICI‐induced irAEs. However, recent lines of evidence suggest that management of irAEs by GCs might be accompanied by impaired antitumor responses and unfavorable effects on ICI treatment and outcomes [30, 31, 32]. GCs inhibit T cell activation and promote immunosuppressive activity of regulatory T cells (Tregs) through the inhibitory molecule, LAG‐3 [24]. We and others have shown that low LAG‐3 levels, particularly on T cells and monocytes, are associated with increased disease activity in arthritis and other inflammatory diseases [33, 34, 35]. This might explain how low LAG‐3 levels can lead to an inflammatory state due to a lack of inhibitory signals. In the previous study, we tested the potential activity of GC and various DMARDs on LAG‐3 expression on synovial and circulating cells in vitro. CG and, to a lesser extent, TNF inhibitors were able to up‐regulate LAG‐3, particularly on synovial monocytes. Moreover, we have demonstrated a positive correlation between GC immunosuppressive activity, reflected by the inhibition of total synovial cell growth and an increased number of synovial monocytes expressing LAG‐3 (CD14^+^LAG‐3^+^ cells). These findings show how GC could attenuate synovial inflammation through upregulation of LAG‐3 on synovial monocytes. In PBMC, GC induced a different pattern from that observed in the synovium by upregulating LAG‐3 on T cells and on monocytes [28]. In the context of rheumatic irEAs induced by ICI therapy, pembrolizumab, an anti‐PD‐1, was found to activate synovial monocytes and increase the production of certain cytokines. A study by Sørensen et al. [36] investigated the involvement of pembrolizumab in the activation of synovial monocytes in vitro in the context of rheumatic irEAs. Their findings showed that pembrolizumab induced the secretion of monocyte chemoattractant‐1 (MCP‐1) that caused activation of intermediate monocytes. This supports the idea that pembrolizumab contributes to monocyte activation in the synovium, which may drive inflammatory arthritis. Since ICI can induce rheumatic irAEs that are commonly managed with GCs or other DMARDs, here we examined whether GC or various DMARDs combined with ICI could induce distinct immune checkpoint molecules in circulating and synovial cells. First, we determined the effect of PEM alone or GC with PEM on inflammatory gene expression and demonstrated that GC+PEM enhanced IL‐10 expression both in PBMCs and SFMCs. We had previously shown that GC induced IL‐10 with concomitant reduction in inflammatory gene expression in SFMCs [37]. Mechanistically, GC (dexamethasone) had been found by others to increase IL‐10 mainly in monocytes but not in lymphocytes [38]. We have now explored the modulation of proinflammatory genes by drug combinations. We observed that PEM alone induced significant IL‐17A expression in PBMCs. Activation of autoreactive T cells is considered the key mediator of ICI‐induced irAEs and may reflect the underlying mechanisms of action of anti‐PD‐1 therapy. In accordance with our results, Bacot et al. [39] demonstrated that anti‐PD‐1 (nivolumab) significantly increased the Th17‐associated cytokines IL‐17A and IL‐21 in healthy donor PBMCs in vitro. Similarly, enhanced IL‐17 production has been shown to occur in patients undergoing treatment with nivolumab [40]. We found that GC+PEM repressed IL‐17 expression as well as that of the other inflammatory genes IFN‐γ and TNF‐α in both PBMCs and SFMCs. Next, PBMCs' proliferation in the presence of the drugs was determined. We observed that PEM alone could not affect the proliferation rate compared with the control with PHA alone. Those results are in accordance with the study of Bacot et al. [39], which observed that anti‐PD‐1 (nivolumab) did not affect the proliferation of healthy donor PBMCs induced by anti‐CD3. For the drug combinations, GC+PEM and MTX+PEM, but not with biologic DMARDs+PEM, significantly inhibited the proliferation. However, simultaneously in the same experiments, GC+PEM induced significant upregulation of CD4^+^LAG‐3^+^ but not of CD4^+^PD‐1^+^ T cells.

In accordance with our results, Acharya et al. [41] observed that GC (dexamethasone) suppressed the proinflammatory cytokines IL‐2, TNF‐α, and IFN‐γ, and induced IL‐10. These activities were accompanied by up‐regulation of PD‐1, TIM‐3, and LAG‐3, but not TIGIT. Two other studies had found that modulation of the same anti‐inflammatory cytokines by GC was accompanied by enhanced expression of PD‐1 on T cells [23, 42]. Both studies claimed that by means of this mechanism, GCs signaling is associated with failure to respond to checkpoint blockades through the promotion of T cell dysfunction that might have important implications for cancer immunotherapy. However, our findings revealed that LAG‐3 induction was not accompanied by PD‐1 induction. In contrast, a study by Okoye et al. [43] determined that concomitant prednisone does not appear to interfere with the function of the immune checkpoint blockade since prednisone, as a single agent or combined with checkpoint inhibitors (anti‐PD‐1 and anti‐CTLA‐4), did not change the expression of PD‐1, CTLA‐4, TIM‐3, and LAG‐3. A possible explanation for the difference between our results and those of Okoye et al. [43] is that those authors induced PBMCs activation with staphylococcal enterotoxin B, anti‐CD3, and anti‐CD28, whereas we used PHA. The choice of mitogen as an activator, might have an influence on the expression of the inhibitory molecules.

We demonstrated that, similar to the effect of combined GC+PEM on up‐regulation of CD4^+^LAG‐3^+^ T cells in PBMCs, this drug combination induced CD14^+^LAG‐3^+^ cells in SFMCs. Synovial CD14^+^ monocytes contributed to the progression of synovitis by differentiating into proinflammatory cytokines (IL‐1β, IL‐6, and TNF‐α), producing cells and inducing osteoclast differentiation, which contributes to bone resorption in the inflammatory milieu [44]. Synovial monocytes constitute a major part of the inflammatory cell population within the synovium [45, 46]. GC may target the predominant monocytes that reside in the synovium and reprogram them into anti‐inflammatory cells expressing LAG‐3.

Moreover, GC+PEM induced an LAG‐3‐dependent pathway since PD‐1 was not promoted by this drug combination. We found that not only GC+PEM induced CD14^+^LAG‐3^+^ cells in SEMCs, but also the biologic DMARD, anti‐TNF, could mediate CD14^+^LAG‐3^+^ cells upregulation, but to a lesser extent than with GC.

There are conflicting lines of evidence on whether GCs interfere with the antitumor responses in cancer patients treated with ICI. Several studies demonstrated that GC does not modify the efficacy of ICI therapy [20, 47, 48], while other studies found that GC with ICI may impact the effectiveness of ICI therapy [17, 49, 50, 51]. However, in clinical practice, both the timing and dosage of GC play a crucial role in modulating ICI efficacy. We demonstrated that LAG‐3‐expressing cells increased gradually in a dose‐dependent manner with GC concentration.

This could suggest that high GC doses may promote LAG‐3 upregulation and could impact the anticancer treatment. Moreover, we have shown that either GC combined with anti‐PD‐1, anti‐CTLA‐4, or chemotherapy drug, cyclophosphamide, induces upregulation of LAG‐3 but not of PD‐1.

In conclusion, GC combined with ICI modulates pro‐ and anti‐inflammatory cytokines that can limit the autoreactive immune response. At the same time, GC with ICI significantly increased the peripheral CD4^+^LAG‐3^+^ T cells and synovial CD14^+^LAG‐3^+^ cells, but not the PD‐1 expression on immune cells. Of note, this activity was exhibited solely by GC but not by other DMARDs, such as MTX. The relevance of this study to the clinical setting highlights the possibility that the GC mechanism of action induces LAG‐3, which might impact the ICI activity.

## 4 Data Limitations and Perspectives

This study has several limitations. First, our in vitro experiments assessed the effects of GC or DMARDs combined with anticancer drugs on the surface expression of LAG‐3 and PD‐1 on immune cells. However, intracellular levels of LAG‐3 and PD‐1 were not evaluated, and it remains unclear whether the tested drugs influence the intracellular accumulation of these molecules. Second, other immune checkpoint markers, such as TIGIT, TIM‐3, and VISTA, are known to play critical roles in regulating immune responses. The potential of the tested drugs to modulate the expression of these additional markers was not explored in this study. Lastly, considering the inherent limitations of in vitro models, future studies are needed to evaluate the drugs’ effects in clinical settings. Specifically, clinical studies involving cancer patients receiving ICI, before, during, and at the end of GC therapy (also GC at different doses), could provide insights into the in vivo activity of these drugs. This would help to clarify how modulation of LAG‐3 and PD‐1 impacts treatment outcomes.

## 5 Material and Methods

### 5.1 Study Population

The study population consisted of 15 healthy donors’ peripheral blood mononuclear cells (PBMCs). Synovial fluid mononuclear cells (SFMCs) were retrieved from 17 psoriatic arthritis (PsA), 11 rheumatoid arthritis (RA), and four peripheral spondyloarthritis (SpA) patients. The demographic characteristics of study participants are shown in Table 1. The patients had to fulfill either the SPARCC, EULAR/ACR, or ASAS classification criteria for PsA, RA, or peripheral SpA [52, 53, 54]. None of them had a cancer diagnosis or had been treated with ICI therapy.

**TABLE 1:** Demographic and clinical characteristics of PsA, RA, and peripheral SpA patients and healthy donors.

Diagnosis	PsA (*n* = 17)	RA (*n* = 11)	Peripheral SpA (*n* = 4)	Healthy donors (*n* = 15)
Age (years), mean ± SE	52.3 ± 3.8	47.5 ± 7.0	51.3 ± 10.4	31.7 ± 2.3
Sex: Female/male	6/11	7/4	2/2	7/8
Disease activity				
TJ, mean ± SE	2.5 ± 0.6	2.3 ± 1.0	7.7 ± 1.2	NA
SJ, mean ± SE	2.0 ± 0.4	2.3 ± 0.8	1.7 ± 0.3	NA
Cells (leukocytes/µL) in SF, mean ± SE	15,500 ± 3600	12,120 ± 3500	18,000 ± 5100	NA
Treatment				NA
Untreated (*n*)	4	3		NA
Steroids (*n*)		2		NA
cDMARDs				NA
MTX	3	3		NA
Salazopyrin (*n*)	3			NA
Leflunomide (*n*)	3	3		NA
bDMARDs	6	1		NA
tsDMARDs	1			NA

Abbreviations: bDMARDs: biologic disease‐modifying anti‐rheumatic drugs; cDMARDs: conventional disease‐modifying anti‐rheumatic drugs; NA: not applicable; PsA: psoriatic arthritis; RA: rheumatoid arthritis; SE: standard error; SF: synovial fluid; SJ: swollen joints; SpA: spondyloarthritis; TJ: tender joints; tsDMARDs: targeted synthetic disease‐modifying anti‐rheumatic drugs.

### 5.2 PBMCs and SFMCs Isolation and Cell Culture Conditions

Blood samples were collected in EDTA tubes from healthy donors and immediately processed for PBMCs’ isolation. SFMCs were obtained from synovial fluid derived from PsA and RA and peripheral SpA patients with at least one swollen joint. The arthrocentesis procedure was performed by an expert rheumatologist at the Department of Rheumatology, Tel‐Aviv Sourasky Medical Center (TASMC), Israel. PBMCs and SFMCs were extracted by density gradient centrifugation with Lymphoprep solution (Axis‐Shield, Oslo, Norway). PBMCs and SFMCs (4 × 10^6^ cells/mL or 0.8 × 10^6^ cells/in 200 µL/well) were cultured in RPMI 1640 medium (Sartorius, Israel) with 10% fetal calf serum (FCS), 2 mM glutamine, 100 U/mL penicillin, and 100 µg/mL streptomycin at 37°C and 5% CO_2_.

### 5.3 In Vitro Co‐Culture with Therapeutic Agents

PBMCs or SFMCs were cultured with the following anticancer drugs: anti‐PD‐1 monoclonal antibody, pembrolizumab (PEM; Keytruda; Merck, Kenilworth, NJ, USA), ipilimumab (IPI; Yervoy, Bristol‐Myers Squibb, NY, USA) at 10 µg/mL, and cyclophosphamide (CY; Endoxan, Baxter Oncology, Halle, Germany) at 10 µM. The immunosuppressive agents: GCs: methylprednisolone acetate (Depo‐Medrol, Pfizer, NY, USA) at 5 µg/mL, in the dose response assays: 0.05, 0.5, 5, and 50 µg/mL were used. Methotrexate (MTX; Medac, Hamburg, Germany) at 0.05 µg/mL. The following biologics were used: anti‐TNF consisting of infliximab (IFX; Remicade, Janssen Biologics, Leiden, the Netherlands) and etanercept (ETN; Enbrel, Pfizer, NY, USA), anti‐IL‐17A, secukinumab (SEC; Cosentyx, Novartis, Basel, Switzerland), and the anti‐IL‐6R, tocilizumab (TCZ; Actemra, Roche, Mannheim, Germany). The cells were cultured for 4–5 days to enable them to respond to the drugs’ stimuli and to acquire distinct immune phenotypes as described in our previous study [28].

### 5.4 Measurement of Lymphocyte Proliferation in Healthy Donors PBMCs via CFSE Assay

Cell proliferation was assessed by means of CFSE. Briefly, healthy donors' PBMCs were pretreated with CFSE before the start of the co‐culture. The cells were suspended in phosphate‐buffered solution (PBS) with 5 µM CFSE (Sigma, St Louis, MO, USA, cat no: 21888), incubated for 15 min at 37°C, and then washed twice with PBS 2% FCS. The cells were cultured at 2 × 10^5^ cells/well in 200 µL volume and suspended in medium with phytohemagglutinin (PHA, Sigma, Cat no: L1688) at a final concentration of 5 µg/mL. The labeled cells underwent cell division following proliferative stimulus (i.e., PHA) in the CFSE‐based assay. The fluorescent dye, CFSE, permits the direct visualization of cell division. The assay controls consisted of PHA‐stimulated PBMCs without therapeutic agents (representing maximal proliferation) and PBMCs without PHA (representing no proliferation). Experimental samples were cultured with PHA in the presence of the indicated drugs. The cells were harvested after 5 days of incubation and analyzed by flow cytometry; a total of 60,000 cells were acquired from each sample.

### 5.5 Flow Cytometry

The cells were stained for 45 min at room temperature with the following anti‐human antibodies: APC‐eFluor780 anti‐CD4 (eBioscience, 47‐0049‐42), Pacific Blue anti‐CD8 (BLG‐301023, BioLegend), FITC‐anti‐CD14 (BLG‐325604), or PE‐anti‐CD14 (BLG‐301850), PerCP‐eFluor 710‐anti‐LAG‐3 (46‐2239‐42, Invitrogen), and PE‐anti‐PD‐1 (12‐2799‐41, Invitrogen). Single‐stained cell control samples were used to set the compensation of the fluorescent signals and nonstained cells. Control samples were used to set the negative population. Cell viability was assessed by dye exclusion using 1 µg/mL propidium iodide (PI) (BLG‐421301) immediately before analysis. Flow cytometry was performed with a FACS Canto II instrument (BD Biosciences), a total of 60,000 cells were acquired from each sample, and the data were analyzed with FlowJo software (Tree Star, Ashland, OR, USA).

### 5.6 Real‐Time PCR

PBMCs and SFMCs were cultured for 72 h with the below‐mentioned drugs for analysis of gene expressions of IFN‐γ, IL‐10, IL‐17A, and TNF‐α. After incubation, the cells were collected, and RNA was isolated by an RNA extraction kit (High Pure RNA Isolation Kit, Roche). For cDNA synthesis, 300 ng total RNA was transcribed to cDNA with a High‐Capacity cDNA Reverse Transcription Kit (Invitrogen, Carlsbad, CA, USA). Gene expression was performed with Fast SYBR Green Master Mix (Applied Biosystems, Foster City, CA, USA) in a StepOnePlus Real‐Time PCR System (Applied Biosystems). All procedures were performed according to the manufacturer's instructions. The following primers for human genes were used: (forward and reverse, respectively): IFNγ 5′‐CTAATTATTCGGTAACTGACTTGA‐3′ and 5′‐ACAGTTCAGCCATCACTTGGA‐3′, IL‐10 5′‐TGGAGGACTTTAAGGGTTAC‐3′ and 5′‐GATGTCTGGGTCTTGGTT‐3′, IL‐17A 5′‐ACCTCATTGGTGTCACTGCTACTG‐3′ and 5′‐TCCTCAGAATTTGGGCATCCT‐3′, TNFα 5′‐CCCAGGGACCTCTCTCTAATC‐3′ and 5′‐ATGGGCTACAGGCTTGTCACT‐3′, GAPDH 5′‐ATGGGGAAGGTGAAGGTCG‐3′ and 5′‐GGGGTCATTGATGGCAACAATA‐3′. The GAPDH levels were used to normalize gene expression levels.

### 5.7 Statistical Analysis

Data are presented as mean ± SE. Nonparametric analyses were performed with the Kruskal–Wallis test followed by Dunn's multiple comparison test. A *p*‐value of < 0.05 was considered statistically significant. All analyses were performed with GraphPad Prism software version 8 (San Diego, CA, USA).

## Author Contributions

Smadar Gertel and Ori Elkayam conceived the original idea, initiated the study, analyzed the data, and supervised the project. Smadar Gertel carried out the experiments and drafted the manuscript. Ari Polachek, Victoria Furer, Tali Ofir Dovrat, Chen Avaky, Adi Broyde, Hila Nochimovitz, and Ori Elkayam contributed to patient recruitment and sample collection, documented the clinical data, treatments, and clinical outcomes, and reviewed and edited the original draft. All authors commented on previous versions of the manuscript, revised and approved the final manuscript.

## Conflicts of Interest

The authors declare no conflicts of interest.

## Ethics Statement

All participants have given written informed consent before enrolment in the study as mandated by the Declaration of Helsinki. The study was carried out between the years 2023 and 2025 and was approved by the Institutional Review Board of Tel‐Aviv Sourasky Medical Center, Israel (0182‐18‐TLV). All procedures were performed in compliance with relevant laws and institutional guidelines. Coded samples were used throughout the study.

## Peer Review

The peer review history for this article is available at https://publons.com/publon/10.1002/eji.70033.

## Supporting information




**Supporting File 1**: eji70033‐sup‐0001‐SuppMat.docx

**Supporting File 2**: eji70033‐sup‐0002‐FigureS1.tif

## Data Availability

The data that support the findings of this study are available from the authors upon reasonable request.
