# Supplementary material for: The Mitigating Effect of Combined Glucocorticoids with Immune Checkpoint Inhibitors on Lymphocyte Activation Gene‐3 and Programmed Death‐1 Expression
Source: Eur J Immunol. 2025 Aug 11;55(8):e70033. doi: 10.1002/eji.70033 (PMC12338123; doi:10.1002/eji.70033)
Supplement: Supplementary file 1 — Supporting File 1: eji70033‐sup‐0001‐SuppMat.docx [file EJI-55-e70033-s001.docx]

**
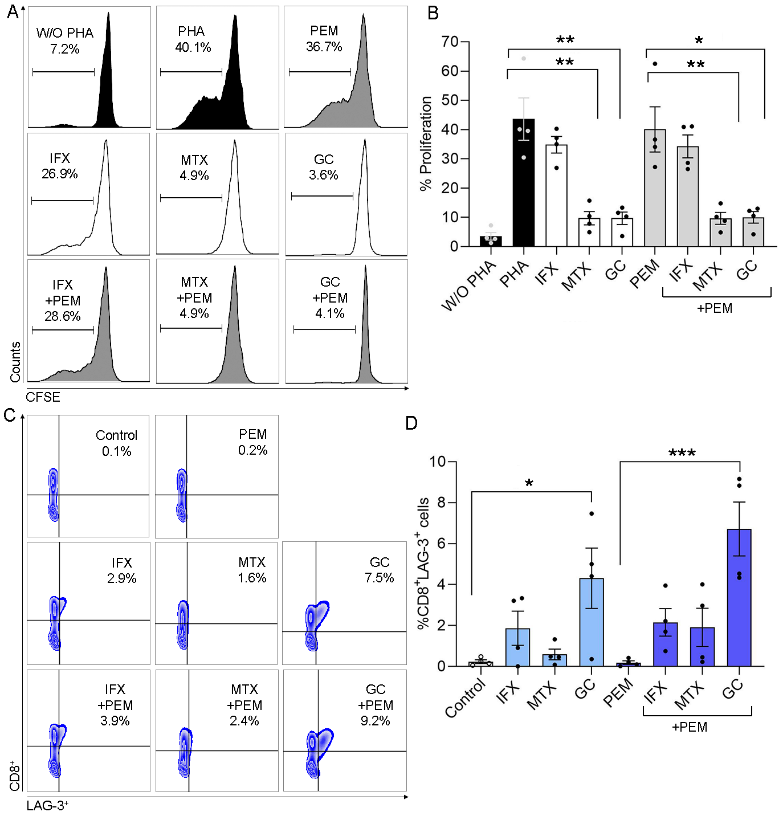
**

**Supplementary Figure 1.** **GC alone or combined with PEM markedly inhibits the PHA-induced proliferation and concomitantly induces CD8^+^LAG-3^+^ T cells**. (A) Healthy donor PBMCs (n=4) were cultured without or in presence of PHA 5 μg/ml or PHA with PEM 10 μg/ml (upper panel). PHA with the drugs as single agents: IFX 10 μg/ml­, MTX 50 ng/ml and GC 5 μg/ml (middle panel)*­,* or the tested drugs combined with PEM (lower panel). Cells were cultured for 5 days and at the end of the culture, were harvested and analyzed by flow cytometry. (A) Representative plots showing examples of the CFSE dilution assay with PBMCs derived from healthy subject. The extent of proliferation is shown at the left side of each plot. (B) Summary of data showing percentage of proliferation. In the same experiments cells were analyzed for frequency of CD8^+^LAG-3^+^ T cells. (C) Healthy donor PBMCs were cultured in the presence of PHA 5 μg/ml without any drug (control) or with PEM 10 μg/ml (upper panel). Comparison of the frequencies of CD8^+^LAG-3^+^ T cells after co-culture with the following drugs as a single agent: IFX*,* MTX, and *GC* (middle panel) or the tested drugs combined with PEM (lower panel). Representative dot plots of CD8^+^LAG-3^+^ T cells*.* The upper right quadrant in each plot shows the percentage of CD8^+^LAG-3^+^ T cells. (D) %CD8^+^LAG-3^+^ T cells in healthy donor PBMCs (n=4). Statistical significance was calculated using the Kruskal-Wallis test with Dunn’s multiple comparison test, *p<0.05, **p<0.03, ***p<0.002 and n.s: non-significant.
