# Supplementary figures and images for: The Mitigating Effect of Combined Glucocorticoids with Immune Checkpoint Inhibitors on Lymphocyte Activation Gene‐3 and Programmed Death‐1 Expression
Source: Eur J Immunol. 2025 Aug 11;55(8):e70033. doi: 10.1002/eji.70033 (PMC12338123; doi:10.1002/eji.70033)

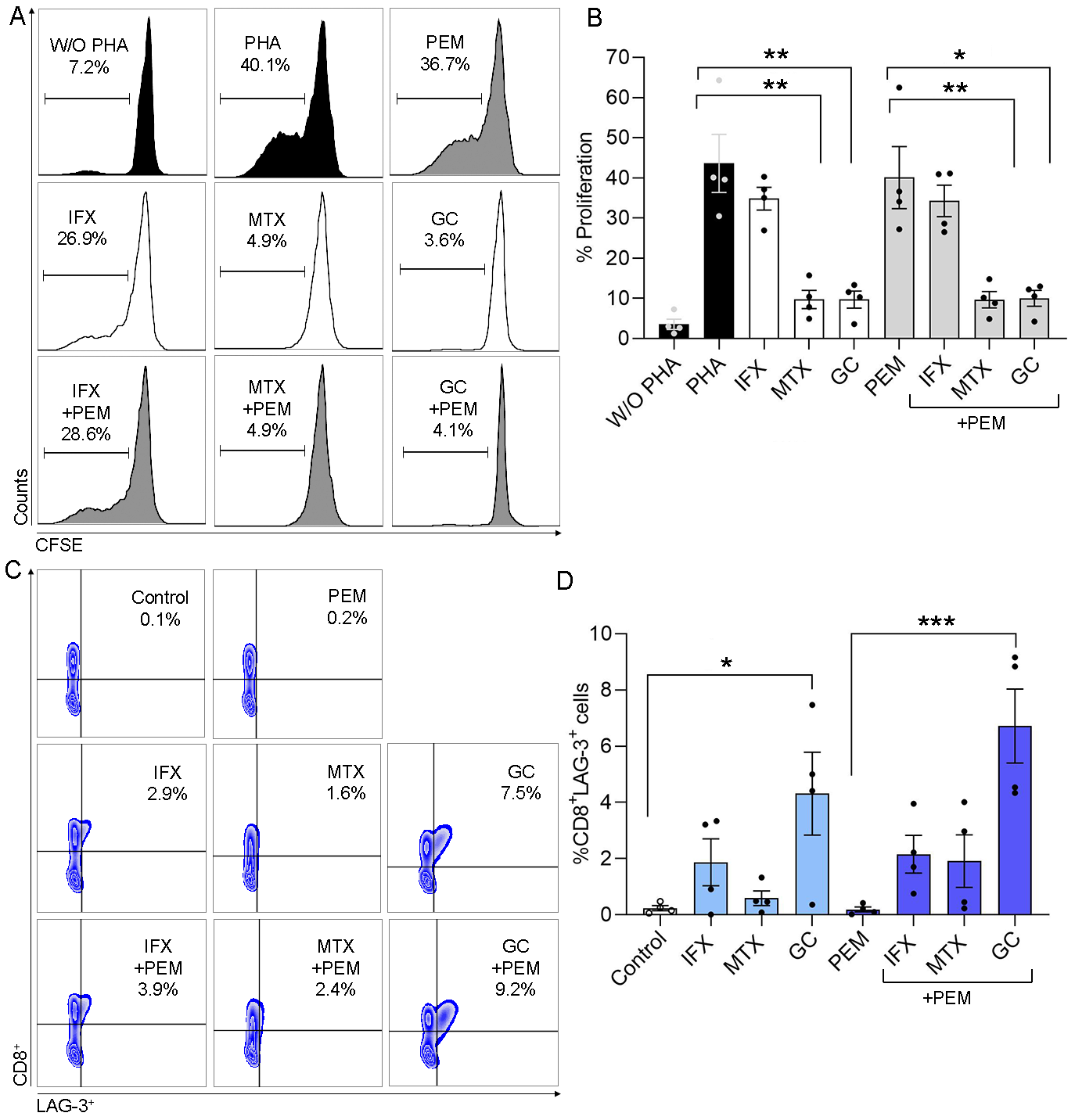

Supplement: Supplementary file 2 — Supporting File 2: eji70033‐sup‐0002‐FigureS1.tif [file EJI-55-e70033-s002.tif]
